# Isopod holobionts as promising models for lignocellulose degradation

**DOI:** 10.1186/s13068-020-01683-2

**Published:** 2020-03-13

**Authors:** Marius Bredon, Benjamin Herran, Joanne Bertaux, Pierre Grève, Bouziane Moumen, Didier Bouchon

**Affiliations:** Laboratoire Ecologie et Biologie des Interactions-UMR CNRS 7267, Ecologie et Biologie des Interactions-Bâtiment B8-B35, Université de Poitiers, 5 rue Albert Turpin, TSA 51106, 86073 Poitiers Cedex 9, France

**Keywords:** Lignocellulose, CAZymes, Shotgun metagenomics, Microbiota, Holobiont, Transcriptomics, Isopods

## Abstract

**Background:**

Isopods have colonized all environments, partly thanks to their ability to decompose the organic matter. Their enzymatic repertoire, as well as the one of their associated microbiota, has contributed to their colonization success. Together, these holobionts have evolved several interesting life history traits to degrade the plant cell walls, mainly composed of lignocellulose. It has been shown that terrestrial isopods achieve lignocellulose degradation thanks to numerous and diverse CAZymes provided by both the host and its microbiota. Nevertheless, the strategies for lignocellulose degradation seem more diversified in isopods, in particular in aquatic species which are the least studied. Isopods could be an interesting source of valuable enzymes for biotechnological industries of biomass conversion.

**Results:**

To provide new features on the lignocellulose degradation in isopod holobionts, shotgun sequencing of 36 metagenomes of digestive and non-digestive tissues was performed from several populations of four aquatic and terrestrial isopod species. Combined to the 15 metagenomes of an additional species from our previous study, as well as the host transcriptomes, this large dataset allowed us to identify the CAZymes in both the host and the associated microbial communities. Analyses revealed the dominance of *Bacteroidetes* and *Proteobacteria* in the five species, covering 36% and 56% of the total bacterial community, respectively. The identification of CAZymes and new enzymatic systems for lignocellulose degradation, such as PULs, cellulosomes and LPMOs, highlights the richness of the strategies used by the isopods and their associated microbiota.

**Conclusions:**

Altogether, our results show that the isopod holobionts are promising models to study lignocellulose degradation. These models can provide new enzymes and relevant lignocellulose-degrading bacteria strains for the biotechnological industries of biomass conversion.

## Background

Microbiota shapes living organisms through complex interactions. Under changing environmental conditions, it may rapidly evolve and influence on the host adaptation and evolution. The microbiota is known to act on animal development, as well as animal health and evolution [1]. Since the “microbiome revolution” of the last 10 years [2], many studies have highlighted the impact of microbiota on the fitness of the host. This change in our vision of organisms led to the recent introduction of the holobiont concept. This concept considers a holobiont as a combination of a host and its associated microbial community, including bacteria, viruses and cellular organisms [3–5]. As a result, a holobiont is an assemblage of species that are metabolically interdependent. Interaction patterns in these systems shape the holobiont’s composition [6], and conversely, bionts (i.e. members of the holobiont) can be agents of developmental plasticity that facilitate the evolution of new phenotypes in animals.

Herbivory, and more specifically lignocellulose degradation, is one of these processes that evolved through symbiont acquisition in many animals [7]. In the context of global climate change, lignocellulose is also an important renewable and sustainable source to produce biofuels and other bioproducts [8]. It seems to be the best alternative to fossil fuels, thus attracting attention from researchers and industrials worldwide. Bacteria and fungi have traditionally been used for research of lignocellulose-degrading enzymes due to their important role in the decomposition of organic matter in ecosystems [9–11]. For most animals, the degradation of lignocellulose involves the cooperation of many bionts to fully achieve its deconstruction [12]. It requires a large number of enzymes that are classified in Carbohydrate Active EnZymes (also called CAZymes) families [13]. CAZymes act on lignocellulose like an enzymatic cocktail; they complement each other and work in synergy to degrade each component of the lignocellulose. Lignocellulose is mainly composed of cellulose, hemicellulose and lignin [14]. CAZymes are thus classified in three classes, depending on the targeted substrate: cellulases, hemicellulases and lignin-modifying enzymes (abbreviated hereafter LME). These three types of enzymes are classified in several CAZy families: the majority of cellulases belong to glycoside hydrolases (GH), the hemicellulases to carbohydrate esterases (CE) and to GHs, and the LME are all classified in auxiliary activities (AA). In addition, recent studies have shown the existence of oxidative cellulases classified in AA families [15]. They break down cellulose with oxidative processes, contrary to the cellulases classified as GHs that hydrolyze cellulose [16, 17]. However, the recalcitrance of lignocellulose, as well as the strong demand of novel enzymes by the industry, imply to explore new models for lignocellulose degradation. Because of the insufficient quantities of enzymes produced by fungi, new research now focuses on bacterial lignocellulose-degrading CAZymes [11]. Expanding bacteria models to the holobiont might enable us to find new strategies for lignocellulose degradation, and thus to respond to the lack of resources for the energetic and chemical industries.

In this context, isopods could be very interesting models to study the lignocellulose degradation in the light of the holobiont concept. Strategies for lignocellulose degradation are different between terrestrial and aquatic isopods [18]. Aquatic isopods have developed specific strategies to degrade the lignocellulose, and for some of them without the help of microbiota [19–21]. The best example is *Limnoria quadripunctata*, a marine wood-boring isopod which feeds on the cellulose without any help from the microbes [21]. Remarkably, whereas hemocyanins conventionally stand as respiratory proteins, those secreted in the hindgut of *L. quadripunctata* modify the lignin and thus enhance the digestibility of cellulose [19]. On the other hand, terrestrial isopods shelter rich and diverse microbial communities in all their tissues [22–24], implying multiple interactions in the holobiont. These communities enable an efficient digestion of the lignocellulose thanks to a complementarity between their CAZome (i.e. CAZyme repertoire) and that of their host [18, 25, 26]. Furthermore, since the land conquest, the CAZomes of terrestrial isopods have been enriched through several gene duplications and horizontal transfers [18]. Strategies for lignocellulose degradation in isopod therefore depend in part on the interactions within the holobiont. Moreover, it has been estimated that herbivory arose independently three times in isopods [27] and promoted Crustacea diversification [28]. As a result, we might suppose that much remains unknown on lignocellulose degradation strategies in isopods.

The main purpose of this study is to provide new features on lignocellulose degradation in the isopod holobionts. To this end, shotgun sequencing of 36 metagenomes was performed from digestive and non-digestive tissues of one freshwater and three terrestrial isopod species from several populations. In addition, we used 15 shotgun metagenomes of our previous study on the pill bug *Armadillidium vulgare* [25]. Combined with the host transcriptomic data, this large dataset enabled us to identify the CAZome of both host and microbiota, and microbial taxa associated with lignocellulose degradation. The comparison of different CAZomes, as well as the identification of CAZymes, PULs (“Polysaccharide Utilization Loci”) and cellulosomes in isopods, highlighted the diversity of strategies for this process in isopods. Altogether, these results show that isopod holobionts are promising models to study lignocellulose degradation and to potentially discover new lignocellulose-degrading CAZymes.

## Results

### Quality of metagenome and transcriptome assemblies

To build the CAZomes of the isopod holobionts, 51 metagenomic samples from digestive and non-digestive tissues of five isopod species were processed from 36 new datasets, along with 15 datasets from our previous study [25]. Samples from different origins enabled us to compare CAZomes of different populations for a single species. These samples represented a total of 5.7 billion reads, assembled into 25 million contigs including 4.7 million contigs > 1 kb length (Additional file 1).

Meanwhile, host transcriptomes were obtained from whole individuals from several populations (for more details, see [18]). Transcriptome assemblies resulted in 40,916 from 143,383 transcripts depending on the species. Assemblies showed an N50 from 1096 bp to 1523 bp depending on the transcriptome (Additional file 2). They displayed a good completeness since more than 95% of the complete genes from the arthropod core genome were present in the *A. vulgare*, *P. dilatatus dilatatus*, *P. dilatatus petiti* and *P. pruinosus* assemblies, and 83% of these genes were present in the *A. aquaticus* assembly (Additional file 2).

### CAZyme identification in isopod holobionts

Both contigs and transcripts from metagenomes and host transcriptomes were subjected to the CAZy database (http://www.cazy.org) to identify CAZomes in isopod holobionts. The use of dbCAN2 for the identification of CAZymes resulted in more stringent criteria than in our previous studies [18, 25] for the identification of CAZymes. In total, 15,834 CAZymes were identified distributed among 201 CAZy families of which 27 were specific to the hosts, 136 were found only in microbiota, and 36 were present in both (Additional files 3, 4, 5). Of these CAZymes, 12,916 belonged to the metagenomes (distributed among 174 families) and 2918 belonged to the host transcriptomes (distributed among 63 families). Eighty-four GH families were identified in the CAZomes of isopod holobionts; they represented the most abundant and diversified families of those CAZomes. GHs are a prominent group of enzymes that hydrolyze the glycosidic bonds, most of cellulases and hemicellulases belong to these families. In the metagenomes, GH13 was the most frequently occurring GH family (799 modules identified in 82,4% of the samples), while GH18 represented the most abundant GH family in transcriptomes (315 modules identified in all samples) (Additional file 3). Then, GTs families were the second most abundant ones with 55 members, of which GT2 was the most abundant GT family in metagenomes (1493 modules identified in 75% of the samples) and GT1 the most abundant in transcriptomes (333 modules identified in all samples). As regards other families, 27 CBMs, 13 CEs, 13 PLs and 9 AAs were identified.

### Lignocellulose-degrading CAZymes

**Fig. 1:**
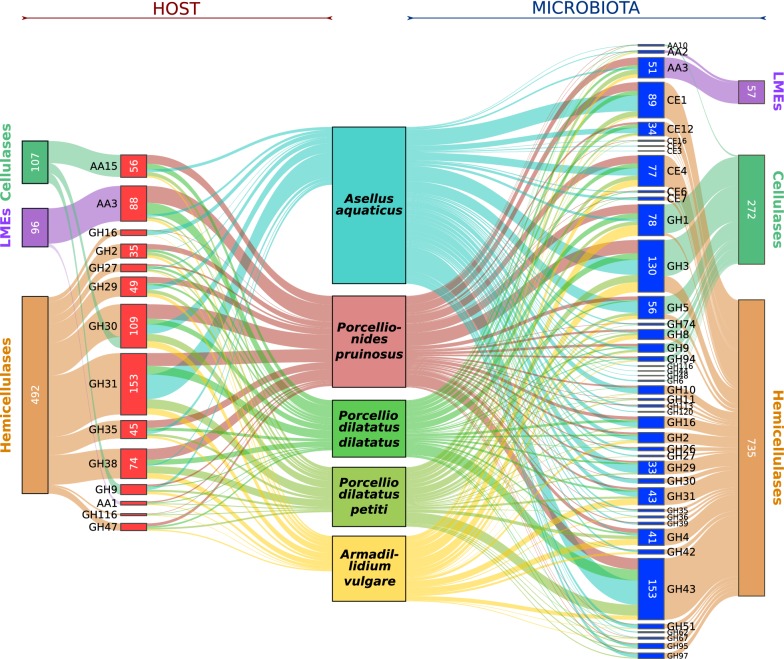
Lignocellulose-degrading CAZymes of isopod holobionts. Numbers represent normalized CAZyme counts identified in the host (red) on the left and microbiota (blue) on the right. The thickness of the connector is proportional with the number of normalized CAZyme counts identified in a given family

Selected CAZymes likely to contribute to the lignocellulose degradation were then examined in depth. Focusing on digestive enzymes in isopod holobionts, only metagenomic samples of caeca and hindgut were considered for the subsequent analyses. In total, 44 lignocellulose-degrading CAZyme families representing 3140 modules were predicted in the metagenomes (Additional file 3), distributed among 33 GH families, eight CE families and three AA families (Fig. 1). Enzymatic activities of 1313 (41.8%) of these modules were predicted by Hotpep (Additional file 6). Among them, 1074 (81.8%) could have a lignocellulosic enzyme activity and 239 (18.2%) potentially act on other substrates (e.g. pectin, chitin).

**Fig. 2:**
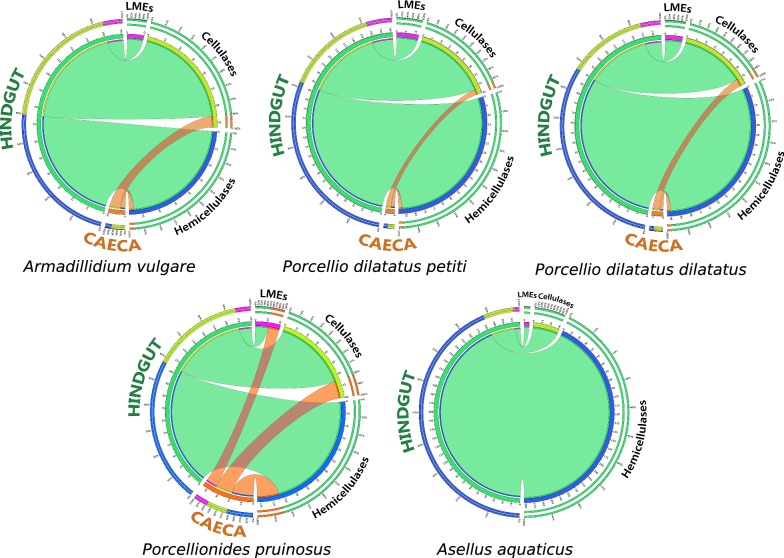
Distribution of lignocellulose-degrading CAZymes from the microbiota in the host digestive tissues. Orange connectors show normalized CAZyme counts identified in caeca and green connectors those identified in hindguts

Most of lignocellulose-degrading CAZymes were identified in the hindgut, especially the LMEs that were absent from the caeca in all species, except *P. pruinosus* (Fig. 2). In all the species, hemicellulases known to degrade a broad range of substrates like xylan, mannan and xyloglucan were the most abundant lignocellulose-degrading CAZyme in the metagenomes with 36 families in total (Fig. 1). This is especially the case for *A. aquaticus* metagenomes in which hemicellulases represent 76% of the identified lignocellulose-degrading CAZyme modules. Concerning cellulases, 12 families were identified in the metagemomes, including seven endocellulases (GH5, GH6, GH8, GH9, GH44, GH48, GH74), three beta-glucosidases (GH1, GH3, GH116), one cellobiose phosphorylase (GH94) and one LPMO (AA10) (Fig. 1). Note that four CAZyme families (GH5, GH8, GH1 and GH3) contain both cellulases and hemicellulases (Fig. 1 and Additional file 6). Finally, two families of LMEs were identified in the metagenomes: one family of laccases (AA1) and one family of cellobiose dehydrogenases (AA3).

Among the 2918 CAZymes belonging to the host transcriptomes, 987 could act as lignocellulose-degrading CAZymes (Additional file 3). The enzyme activity of 567 (57.45%) of those CAZymes was predicted using Hotpep, 555 (97.9%) of which could degrade the lignocellulose (Additional file 6). They were distributed among 14 CAZy families including 11 GHs and three AAs (Fig. 1). Host lignocellulose-degrading CAZymes were thus three times less diversified and abundant than in the microbiota, and only four were specific: GH47, GH38, AA15, and AA1. Three cellulases were identified in isopod hosts: two cellulases widespread in crustaceans, belonging to the GH9 and GH30 families [20, 29, 30], and an LPMO, belonging to the AA15 family, that was recently identified in arthropods [17, 18]. Concerning LMEs, a few laccases belonging to the AA1 family were identified in terrestrial isopods but not in the aquatic species, and some AA3s were found in the five species (Fig. 1). Finally, just like for the metagenomes, hemicellulases were the most diversified CAZymes in the host transcriptomes with 11 families identified. Compared to the terrestrial species, the microbiota of the freshwater isopod *A. aquaticus* seems to contribute more than the host to lignocellulose degradation (Fig. 1). Indeed, only 11 lignocellulose-degrading CAZymes were identified from the transcriptome, whereas 43 lignocellulose-degrading CAZymes were identified from the metagenome, which is 1.5 times more than in the metagenomes of terrestrial species (containing 28 lignocellulose-degrading CAZymes on average).

### Taxonomic origin of the CAZymes from microbiota

**Fig. 3:**
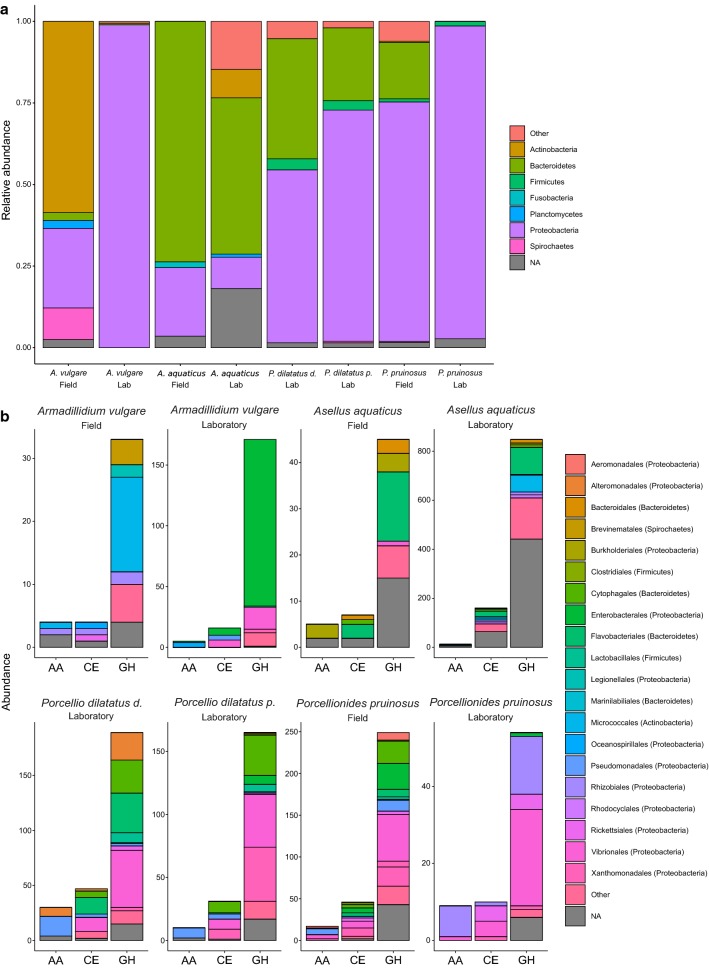
Compositions of the bacterial community associated with the lignocellulose degradation at the level of the phylum (**a**) and of the order (**b**) according to CAZy classes

**Fig. 4:**
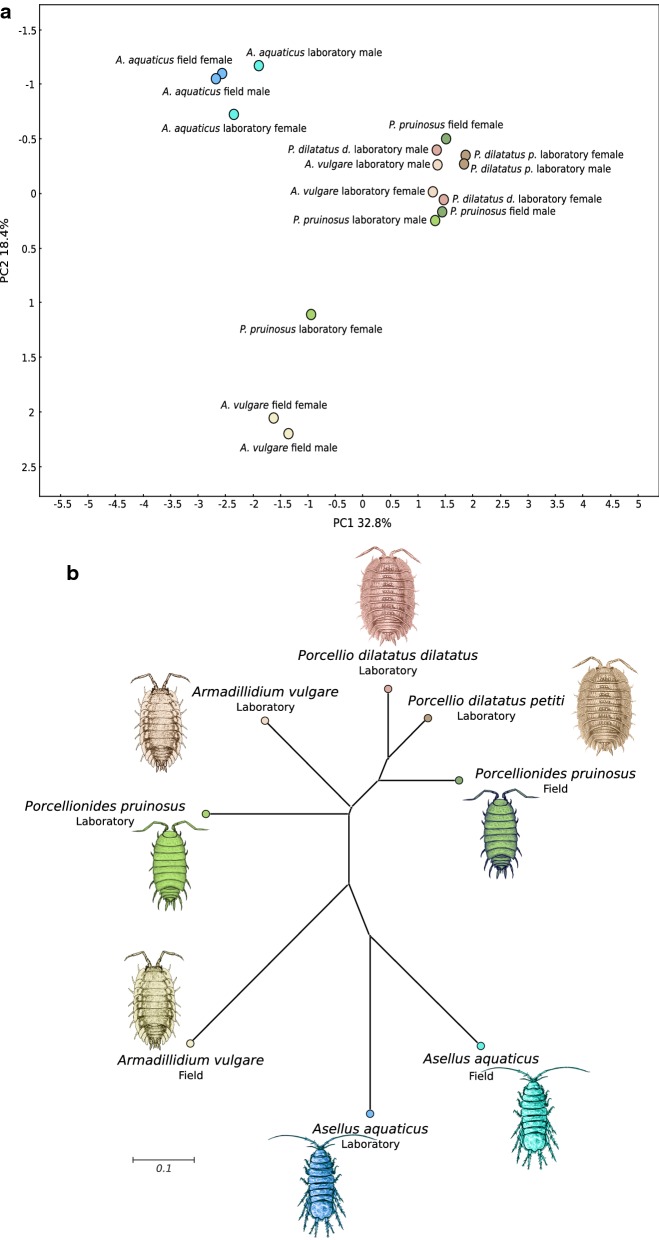
Comparative analysis of bacterial communities associated with the lignocellulose degradation in isopods, considering (**a**) or not (**b**) the effect of sex. **a** A principal component analysis (PCA) plot of the bacterial counts, at the family level, that characterize the bacterial community from each studied species depending of its origin and sex (51.2% of the information was extracted from the two principal components PC1 and PC2). Each dot represents the taxonomic composition of a metagenome and each color represents the host species and its origin (laboratory or field). **b** Phylogenetic tree of the bacterial communities of the studied isopods. All branches are drawn to scale as indicated by the scale bar

To identify the microbial communities associated to lignocellulose degradation, similarity searches of the predicted CAZymes from the metagenomes were performed against the NCBI Non-Redundant Protein database. Proteobacteria and Bacteroidetes were the most represented bacterial phyla, accounting for 56% and 36% of the identified CAZymes, respectively (Fig. 3a). The microbial communities were rich and highly diversified in all host species. Indeed, 41 bacterial orders were found in all microbiota (Fig. 3b). The taxonomic origin of the microbial CAZomes was highly different from one host to another (Figs. 3, 4), without apparent sex effect except for *P. pruinosus* (Fig. 4a). Moreover, the microbial CAZomes were shaped by the environment, as for a given species these communities differed according to the host origin (field or laboratory) (Fig. 4). Compared to terrestrial isopods, the microbiota of *A. aquaticus* included more Bacteroidetes and less Proteobacteria (Fig. 3a). Furthermore, there was a high percentage of non-identified bacteria, notably for the communities that encode GH families in the *A. aquaticus* laboratory lineage, where more than half of bacterial families were unknown (Fig. 3b).

Flavobacteriales (Bacteroidetes), Micrococcales (Actinobacteria) and Burkholderiales (Proteobacteria) were the largest contributors to lignocellulose-degrading CAZymes, especially GH, CE and AA families, in both populations of *A. aquaticus* (Fig. 3). In the terrestrial host species, Vibrionales (Proteobacteria) encoded many lignocellulose-degrading CAZymes belonging to GH families in all hosts, as well as AA and CE families in some of them. Cytophagales (Bacteroidetes) were abundant in *P. pruinosus* (field population), *P. dilatatus dilatatus* and *P. dilatatus petiti.* Likewise, Rhizobiales (Proteobacteria) encoded the most part of lignocellulose-degrading CAZymes in *P. pruinosus* originating from laboratory. Finally, among other bacterial families, Xanthomonadales (Proteobacteria), Enterobacteriales (Proteobacteria) and Flavobacteriales (Bacteroidetes) were also important contributors to lignocellulose degradation in some terrestrial isopod species.

### Identification of PULs and cellulosomes in the microbiota

**Fig. 5:**
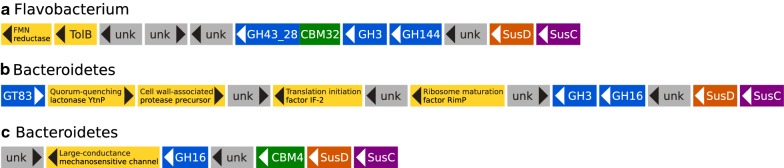
Gene organization in PULs identified in the metagenomes of *A. aquaticus.* PULs were assigned to unclassified Flavobacterium and Bacteroidetes. In addition to genes encoding CAZymes, various other genes and unknown proteins (abbreviated “unk” in the figure) were also present

Potential PULs were screened by searching within contigs for both gene markers of PULs: *SusC* and *SusD*. The *SusC* or/and the *SusD* conserved domains were identified in 5089 contigs. Among them, only 37 contigs encoded one sequential pair of *susC* and *susD* (Additional file 7). They were found in all species except *A. vulgare* and the laboratory lineage of *P. pruinosus*. Unsurprisingly, all these contigs were assigned to Bacteroidetes species. In the metagenomes of *A. aquaticus*, three contigs harboring *Sus* genes were of interest as they also encoded CAZymes which might be involved in lignocellulose degradation (Fig. 5). Indeed, they could act in the breakdown of hemicellulosic substrates thanks to their GH3, GH16 and GH43 enzymes. These PULs exhibited a gene organization different from those referenced in the PUL DataBase [31].

Potential cellulosomes were predicted by searching cohesin or dockerin modules within contigs. In total, 835 dockerin modules and 65 cohesin modules were identified in 874 contigs of the metagenomes (Additional files 8, 9). In addition, 163 contigs harbored an SLH domain (Additional files 8, 9), an anchoring module that helps to bind the cellulosome to the cell surface. The presence of a dockerin domain in 30 CAZyme genes provided evidence for active cellulosomes in microbiota (Table 1). Several of those CAZymes are known to deconstruct lignocellulose: CE1, CE3, GH2, GH3, GH5, GH26, GH35, GH43 and GH44. In addition, one GT (GT102), three CBMs (CBM32, CBM35, CBM38), one PL (PL9), nine other GHs (GH18, GH33, GH50, GH93, GH99, GH108, GH130, GH135, GH136) and one other CE (CE10) were also associated with dockerin domains. The taxonomic assignation of these contigs showed that potential cellulosomes were carried out by bacteria belonging to Bacteroidetes, Planctomycetes, Armatimonadetes, Rhizobiales and several unknown bacteria (Table 1).

**Table 1:** CAZymes containing dockerin domains

Host	Origin	Gender	Tissue	Assignation	CAZymes
*A. aquaticus*	Field	Female	Nd.^a^ tissues	Bacteria	GH35
*A. aquaticus*	Field	Female	Nd.^a^ tissues	Bacteroidetes	GH130
*A. aquaticus*	Field	Female	Nd.^a^ tissues	Cytophagales	GH35
*A. aquaticus*	Field	Male	Nd.^a^ tissues	Bacteria	GH130
*A. aquaticus*	Field	Male	Nd.^a^ tissues	Cytophagales	GH35
*A. aquaticus*	Laboratory	Female	Hindgut	Bacteria	GH3
*A. aquaticus*	Laboratory	Male	Hindgut	Armatimonadetes	CE10
*A. aquaticus*	Laboratory	Male	Hindgut	Bacteria	GH18
*A. aquaticus*	Laboratory	Male	Hindgut	Bacteria	GH136
*A. aquaticus*	Laboratory	Male	Hindgut	Bacteria	GH2
*A. aquaticus*	Laboratory	Male	Hindgut	Bacteria	GH50
*A. aquaticus*	Laboratory	Male	Hindgut	Bacteria	PL9_2
*A. aquaticus*	Laboratory	Male	Hindgut	Bacteria	CE1
*A. aquaticus*	Laboratory	Male	Hindgut	Bacteria	GH5_13
*A. aquaticus*	Laboratory	Male	Hindgut	Bacteria	GH93
*A. aquaticus*	Laboratory	Male	Hindgut	Bacteria	GH99
*A. aquaticus*	Laboratory	Male	Hindgut	Bacteria	GH135
*A. aquaticus*	Laboratory	Male	Hindgut	Phycisphaerae	GH99
*A. aquaticus*	Laboratory	Male	Hindgut	Planctomycetes	GH44
*A. aquaticus*	Laboratory	Male	Hindgut	Planctomycetes	GH26 + CBM35
*A. aquaticus*	Laboratory	Male	Hindgut	Planctomycetes	GH18
*A. aquaticus*	Laboratory	Male	Hindgut	Planctomycetes	GH43_5
*A. aquaticus*	Laboratory	Male	Hindgut	Planctomycetes	CE1
*A. aquaticus*	Field	Female	Nd.^a^ tissues	Polynucleobacter	GT102
*P. dilatatus d.*	Laboratory	Male	Nd.^a^ tissues	Algoriphagus	GH33
*P. dilatatus p.*	Laboratory	Female	Hindgut	Algoriphagus	CBM38
*P. dilatatus p.*	Laboratory	Female	Nd.^a^ tissues	Bacteroidetes	CBM32
*P. pruinosus*	Field	Female	Hindgut	Bacteroidetes	CE3
*P. pruinosus*	Field	Female	Hindgut	Rhizobiales	GH108
*P. pruinosus*	Laboratory	Female	Caeca	Rhizobiales	CE10

Assignation corresponds to the closest bacterial taxon possible as predicted by CAT-BAT

^a^Nd. non-digestive tissues

## Discussion

We investigated the repertoire for lignocellulose degradation on the scale of the holobiont in five isopod species, aquatic and terrestrial, highlighting that host and microbiota complement their CAZomes to achieve effective lignocellulose deconstruction. Despite using more stringent criteria than in our previous studies [18, 25], we found a great diversity of CAZymes in all isopod holobionts. We also showed that highly different host-associated bacterial communities occurred in the terrestrial species and even within species, revealing a functional redundancy in lignocellulose degradation and as regards complementarity with the host’s repertoire. In addition, we highlighted the potential involvement of microbial cellulosomes, as well as the presence of PUL systems in isopods. Isopods therefore represent an unexploited wealth of lignocellulose-degrading enzymes and natural nanomachines.

The microbiota had a prominent weight in the lignocellulose-degradation repertoire of the five isopod holobionts: the microbial lignocellulose-degrading CAZymes exceeded by three times those of the hosts. A large part was not represented in the hosts: accordingly, the diverse microbial CAZymes might complete the CAZome of the host for efficient lignocellulose degradation. Considering that host CAZymes are primarily produced in the caeca [25], the production of CAZymes in the hindgut by microbiota contributes to sequential biomass degradation in the digestive organs. Spatially, the digestion of lignocellulose is therefore performed along the different parts of the digestive tract thanks to the successive intervention of host and microbial enzymes from the foregut where the caeca (or digestive glands) open to the posterior part of the hindgut [26, 32, 33].

Hemicellulases are the most numerous lignocellulose-degrading CAZymes in isopod holobionts. Since hemicellulose composition varies from one plant to another, and even from one plant tissue to another [16], organisms require large repertoires of hemicellulases to be able to degrade these complex structures. As decomposers, isopods have to deal with a wide variety of foods. The diversity of hemicellulases we recorded in both host and microbiota might be an adaptive response of isopod holobionts to the complex composition of hemicellulose. Several types of cellulases belonging to endocellulases and beta-glucosidases were also identified in both host and microbiota. In contrast, no exocellulases were found in the five studied isopods. Whereas they are required by fungi to achieve the cellulose degradation [34], they are rarely found in animals, among which a few crustaceans, including five isopod species not corresponding to those presently studied [13, 18, 30, 35, 36]. Contrary to termites where exocellulases are provided by their symbionts [37–39], isopods may degrade the cellulose thanks to the abundance and diversity of other kinds of cellulases provided by their microbiota. Furthermore, we have identified oxidative cellulases (LPMO) belonging to AA15 family in host transcriptomes and LPMO belonging to AA10 in the metagenomes of *A. aquaticus*, *P. pruinosus* and *P. dilatatus p.*, suggesting the existence of alternative strategies for cellulose degradation in isopod holobionts. Finally, isopod hosts might be key players of lignin modification for a better exploitation of hemicellulose and cellulose. Numerous cellobiose dehydrogenases (CDH) belonging to the AA3 family and laccases belonging to AA1 family were found in the four terrestrial host species. Laccases are among the most important LME in wood-destroying microorganisms [40, 41] but CDHs are not widespread in arthropods and are even absent in insects [18]. In addition, in the five isopod species, the microbiota provides manganese peroxidases belonging to the AA2 family, as well as some AA3 enzymes. Once again, the host–microbiota cooperation could permit isopod to efficiently breakdown the lignin allowing access to other lignocellulose components.

It is interesting to note that host–microbiota contribution to lignocellulose degradation appeared different in the freshwater isopod *A. aquaticus* compared to the terrestrial ones. The host CAZome of *A. aquaticus* is less expanded than those of terrestrial species [18]. However, its microbial CAZome comprised 43 different lignocellulose-degrading CAZymes, which is 1.5 times more than that of terrestrial species. Moreover, the freshwater isopod did not have laccases for lignin degradation, and most of the LMEs seem to be produced by its microbiota, as described in accordance with Zimmer and Bartholmé [42]. Similarly, the CAZome of its microbiota contained a significant number of hemicellulases, which could allow *A. aquaticus* to degrade the wide variety of consumed plants and fungi [43]. *Asellus aquaticus* would thus compensate its small enzymatic repertoire thanks to its microbiota which would then provide the necessary CAZymes to achieve food digestion.

We also showed that the microbiota could use cellulosomes and PULs to improve lignocellulose degradation. To our knowledge, this is the first evidence of the presence of cellulosomes and PULs in isopods. Only Zimmer [26] has suspected the presence of cellulosomes in bacteria from the hindgut of terrestrial isopods. Cellulosomes are described as “one nature’s most elaborate and highly efficient nanomachines” [44]. They are multiprotein complexes where associated enzymes collaborate to degrade cellulose and hemicellulose. Each enzyme is specifically regulated allowing for a sequential intervention, thus avoiding any competitive interactions [45]. We have identified potential cellulosomes in bacteria mainly found in the hindgut of both aquatic and terrestrial isopod hosts. Many CAZymes associated with a predicted dockerin domain are known to degrade cellulose and hemicellulose, indicating an enhanced ability for the isopod microbiota to degrade lignocellulose through cellulosomes. Identified cohesin and dockerin modules belong to several bacteria species, suggesting inter-species and intra-species cohesin–dockerin interactions [46–48]. PULs are other complexes first described in Bacteroidetes genomes for lignocellulose and other carbohydrates degradation [49]. They are organized in several co-regulated and co-localized genes encoding CAZymes, sensing proteins, binding proteins, and transporters [50]. We found several candidate contigs harboring PUL genes markers *SusC* or/and *SusD*. Among them, we have predicted several PULs involved in hemicellulose degradation and affiliated to unclassified Bacteroidetes bacteria. Nevertheless, there are probably a great number of fragmented PULs in our metagenomes due to the high proportion of *SusC* and *SusD* orphan domains identified in the contigs. Isopod holobionts are therefore good candidates for research on cellulosomes and PULs, and for the discovery of new bacteria taxa encoding these complexes.

Microbial communities linked to lignocellulose degradation are highly diversified in all host species. Most of the 43 identified bacteria families belong to Actinobacteria, Bacteroidetes and Proteobacteria phyla, where many species are known to produce CAZymes [13]. Bacterial communities associated to lignocellulose degradation are different not only across species, but also between populations in a single species. The isopod microbiota is mostly composed of environmental bacteria and depends on several factors like environmental conditions, sex and season [22, 23, 51, 52]; intra- and inter-host diversity is thus very labile. Yet, as it has been observed from previous studies in *A. vulgare* [24, 25], there is a probable functional redundancy for the lignocellulose degradation between isopod microbiota, in particular in terrestrial host species. This functional redundancy directly reflects the holobiont concept, which considers that each biont is selected through metabolic and developmental interactions that take place in the holobiont [6]. The lignocellulose degradation is therefore an important process that shapes and drives the isopod holobiont composition, selecting the function over the individual. From this point of view, isopods differ from other well-known lignocellulose decomposers (e.g. ruminants, termites, etc.), where transient microorganisms have evolved into heritable symbionts and thus promoting the evolution of herbivory [7].

## Conclusion

As part of the holobiont concept, isopods are excellent models to study lignocellulose degradation. First, they harbor diverse and rich microbial communities in their digestive tissues, likely providing them with complementary lignocellulose-degrading CAZymes. Lignocellulose degradation is therefore possible thanks to multiple interactions between the host and its microbial bionts. Second, strategies for lignocellulose digestion vary across isopod species. In the freshwater isopod *A. aquaticus*, the contribution of the microbiota for this process is much more important than in terrestrial species. On the contrary, marine isopods of the *Limnoria* genus degrade the lignocellulose without the help of any microbiota [19]. While the latter use their hemocyanins to facilitate lignocellulose digestion, terrestrial and freshwater isopod could use PUL and cellulosome systems from their microbiota, as well as specific enzymes like LPMOs to improve their digestion of lignocellulose. It is very likely that many strategies remain to be discovered in isopods, especially in marine ones, which constitute the most abundant and yet the least studied group of species. Isopods are therefore promising models for the biotechnological industries for biomass conversion. The discovery of novel CAZymes and relevant lignocellulose-degrading bacteria strains in isopod holobionts would help promote new sustainable methods and tools to replace fossil fuels.

## Methods

### Biological samples

Metagenomic data were generated from laboratory lineages of the freshwater isopod *Asellus aquaticus*, and the terrestrial isopods *Porcellionides pruinosus*, *Porcellio dilatatus dilatatus* and *Porcellio dilatatus petiti*. Field populations of *Asellus aquaticus* from the Pinail nature reserve (France, 46° 42′ 2.698″ N, 0° 31′ 13.378″ E) and *Porcellionides pruinosus* from Nouaillé-Maupertuis (France, 46 30′ 34″ N, 0° 24′ 54″ E) were also collected in July 2017 (Additional file 1). Those individuals were kept until dissection (within 2 days after collection) in plastic boxes with water or soil from their respective sampling sites. *Armadillidium vulgare* data were issued from our previous metagenomic study [25].

### Metagenomics: DNA extraction and sequencing

Prior to dissection, all individuals were surface-sterilized using sodium hypochlorite. Tissues were then dissected out using sterilized instruments. All tissues were rinsed in Ringer solution to avoid cross-contamination between tissues. Caeca and hindguts (with their contents) were kept as separate samples, and the remaining tissues (i.e. nerve cords, gonads and fat tissues) were pooled. All samples were homogenized in extraction buffer, and total DNA was purified using phenol–chloroform [53]. Equimolar amounts of DNA from seven biological replicates (except for females of *A. aquaticus* from the Pinail nature reserve for which only six individuals could be sampled) of the same tissue and sample type (i.e. origin and sex) were pooled. Then, prokaryotic DNA was enriched twice in each pool using the NEBNext^®^ Microbiome DNA Enrichment kit (New England Biolabs) according to the manufacturer’s instructions. This resulted in 36 shotgun metagenomic libraries which were sequenced on an Illumina HiSeq 4000 by GenoScreen (Lille, France), generating 2 × 150 bp pair-end reads (Additional file 1).

### Metagenomic shotgun assembly

Read quality was checked with FastQC (version 0.11.2; http://www.bioinformatics.babraham.ac.uk/projects/fastqc) and removal of sequencing adaptors and low quality bases was performed with Trimmomatic (version 0.32; [54]). Trimmed reads shorter than 50 bp were discarded. To identify and filter rRNA reads, SortMeRNA was used with an *E* value cut-off of 1e-20 (version 2.1; [55]). To discard host reads, remaining reads were mapped using BOWTIE2 (version 2.3.4.3; [56]) against a custom isopod database comprising all isopod sequences from the Nucleotide NCBI database and unpublished isopod sequences from our laboratory. Non-mapped reads were then assembled with the MEGAHIT software (version 1.0.3; [57]) using the following parameters: –min-count 2 –k-min 21 –k-max 127 –k-steps 1. Assembly qualities were checked using Blobtools (version 1.0; [58]).

To ensure that all host sequences were filtered, two control steps were performed. (1) ORFs from contigs were predicted using Prodigal (version 2.60; [59]) with “meta” parameter, and they were compared against the Non-Redundant Protein database (December 1, 2018) using BLASTX [60] with an *E* value cut-off of 0.0001. The BLAST outputs were then imported into MEGAN6 software (version 6.15; [61]) for taxonomic assignment based on the lowest common ancestor (LCA) algorithm using the NCBI taxonomy database. All contigs associated to an ORF assigned to eukaryotes were discarded. (2) Then, the remaining contigs were run through the CAT-BAT pipeline (version v4.6; [62]) for taxonomic classification and those that were assigned to eukaryotes were discarded.

### Host transcriptomes

Host transcriptomes of whole individuals of *P. dilatatus dilatatus*, *P. dilatatus petiti*, *P. pruinosus*, *A. aquaticus* and *A. vulgare* were the same as those used in Bredon et al. [18]. In brief, reads were trimmed using Trimmomatic (version 0.32; [54]), transcriptome assemblies were performed with IDBA-TRAN [63] with default parameters, then transcripts were clustered with ≥ 95% identity using CD-HIT-EST (version 4.6; [64]) and ORFs were predicted using Transdecoder (version 3.0.1; https://transdecoder.github.io/). The completeness of the resulting assemblies was assessed with BUSCO (version 3.0.1; [65]) referring to core arthropod genes.

### Carbohydrate-Active enZyme annotation

CAZymes were identified using the Carbohydrate Active enZymes (CAZy) database [13]. dbCAN2 [66] was used to identify CAZy families (i.e. Glycoside Hydrolases (GHs), Glycosyl Transferases (GTs), Polysaccharide Lyases (PLs), Carbohydrate Esterases (CEs), Auxiliary Activities (AAs) and Carbohydrate-Binding Modules (CBMs)) from the previously filtered ORFs from the metagenomes and transcriptomes. The software integrates three tools for CAZymes annotation: (1) HMMER (version 3.2.1; [67]) that uses the dbCAN CAZyme domain HMM database [68] for domain predictions, (2) DIAMOND (version 0.9.24; [69]) for sequence comparisons against a custom pre-annotated CAZyme sequence database, and (3) HOTPEP [70] that performs searches against a conserved CAZyme short peptide database. dbCAN2 was run with the following parameter: –dia_eval 1e-50. In a conservative manner, only CAZymes that were predicted by the three tools were kept for the following analyses.

As recommended by dbCAN2 authors, CAZyme counts (note that “CAZyme” refers to functional modules or domains, not genes) resulting from dbCAN assignment using HMMER tool were considered for the following analyses. For comparative analyses, CAZyme counts were normalized to even out the heterogeneity arising for differential library sizes. For each sample, CAZyme counts were divided by the number of ORFs in the metagenome or transcriptome of interest to calculate the relative abundance for each CAZyme family. Then, the normalized count of each family in each metagenome or transcriptome was calculated by multiplying the relative abundance by the lowest number of ORFs identified in corresponding datasets: 202,349 (i.e. male tissues of *P. dilatatus dilatatus*) for the newly sequenced metagenomes, 22,641 (i.e. female tissues of *A. vulgare* from the field) for *A. vulgare* metagenomes of our previous study and 19,473 (i.e. *A. aquaticus* transcriptome) for host transcriptomes.

All CAZy families known to potentially contribute to lignocellulose degradation were then selected for further analysis. However, a single CAZy family can bring together enzymes involved in a large variety of carbohydrate-modifying activities, including lignocellulose degradation. For that reason, the enzymatic activities of the CAZymes belonging to those families were predicted using Hotpep [70] in order to confirm their implication in lignocellulose degradation. When Hotpep could not predict the function of the CAZymes, we considered their most common activity reported in the CAZy database [13].

### Community profiling

To identify microbial communities encoding CAZymes, ORFs annotated as CAZymes were compared with the Non-Redundant Protein database (December 1, 2018) using BLASTP [60]. An *E* value cut-off of 0.0001 was used and the top five hits were kept. MEGAN6 software (version 6.15; [61]) was then used for taxonomic assignation of ORFs using the NCBI taxonomy database, and to construct principal component analysis (PCA) and phylogenic tree. Results were visualized using the Phyloseq R package [71].

### PUL and cellulosome identification

PULs consist of co-localized and co-regulated genes organized around an *SusC*–*SusD* gene pair and encoding proteins that degrade complex carbohydrates. They might play an important role in the breakdown of lignocellulose in Bacteroidetes species [49]. To identify potential PULs in microbiota, ORFs of metagenomes were first compared to the Pfam database (version 32.0; [72]) using hmm-search (version 3.2.1; [67]) with an *E* value cut-off of 0.0001 to identify conserved domains. Then, contigs encoding PUL gene markers [*SusD* like proteins (PF07980) and TonB-dependent receptor/*SusC* like proteins (PF00593)] were extracted and those encoding one sequential pair of *susC* and *susD* were kept for the following analyses. These sequences were subjected to CAT-BAT for taxonomic assignation, dbCAN2 for CAZyme annotation and Prokka (version 1.9; [73]) for gene annotation.

Cellulosomes are multi-enzyme lignocellulosic systems organized around a scaffolding and attached to bacterial cells [44]. Enzymes bind to the scaffolding thanks to interactions among cohesin and dockerin modules. To identify cellulosome systems in microbiota, we searched for cohesin Pfam domain (PF00963) and dockerin Pfam domain (PF00963) in the conserved domains predicted above, as well as for S-layer homology domain (SLH; PF00395). Then, contigs encoding those modules were subjected to CAT-BAT for taxonomic assignation and dbCAN2 for CAZyme annotation.

## Supplementary information

**Additional file 1.** Metrics of the metagenomic samples and resulting assemblies.

**Additional file 2.** Metrics of the transcriptomic samples and resulting transcriptomes.

**Additional file 3.** List of CAZymes identified in the metagenomes and transcriptomes assemblies.

**Additional file 4.** Protein sequences of CAZyme genes identified in the metagenome assemblies.

**Additional file 5.** Protein sequences of CAZyme genes identified in the transcriptome assemblies.

**Additional file 6.** List of predicted enzymatic functions of CAZymes identified in metagenomes and host transcriptomes.

**Additional file 7.** List of PULs identified in the metagenomes.

**Additional file 8.** List of cohesin, dockerin and SLH modules identified in metagenomes.

**Additional file 9. ** Contig sequences that contain dockerin, cohesin and SLH modules.

## Data Availability

Reads used for transcriptomes assemblies are available from the NCBI Sequence Read Archive under accession numbers provided in Additional file 1. Identified CAZymes are provided in FASTA format in Additional files 4 and 5.

## Abbreviations

AAs: Auxiliary activities
CAZome: Cazyme repertoire
CAZymes: Carbohydrate-active enzymes
CBMs: Carbohydrate-binding modules
CDHs: Cellobiose dehydrogenases
cDNA: Complementary deoxyribonucleic acid
CEs: Carbohydrate esterases
DNA: Deoxyribonucleic acid
GHs: Glycoside hydrolases
GTs: Glycosyltransferases
LMEs: Lignin-modifying enzymes
LPMOs: Lytic polysaccharide mono-oxygenases
NCBI: The National Center for Biotechnology Information
ORF: Open reading frame
PCA: Principal component analysis
Pfam: Protein families
PLs: Polysaccharide lyases
PULs: Polysaccharide utilization loci
RNA: Ribonucleic acid
rRNA: Ribosomal ribonucleic acid
